# Vision-Based Target Finding and Inspection of a Ground Target Using a Multirotor UAV System

**DOI:** 10.3390/s17122929

**Published:** 2017-12-17

**Authors:** Ajmal Hinas, Jonathan M. Roberts, Felipe Gonzalez

**Affiliations:** Robotics and autonomous systems, Queensland University of Technology (QUT), Brisbane City QLD 4000, Australia; Jonathan.roberts@qut.edu.au (J.M.R.); felipe.gonzalez@qut.edu.au (F.G.)

**Keywords:** unmanned aerial vehicles, vision-based navigation, vision and action, OODA, remote sensing, inspection, target detection

## Abstract

In this paper, a system that uses an algorithm for target detection and navigation and a multirotor Unmanned Aerial Vehicle (UAV) for finding a ground target and inspecting it closely is presented. The system can also be used for accurate and safe delivery of payloads or spot spraying applications in site-specific crop management. A downward-looking camera attached to a multirotor is used to find the target on the ground. The UAV descends to the target and hovers above the target for a few seconds to inspect the target. A high-level decision algorithm based on an OODA (observe, orient, decide, and act) loop was developed as a solution to address the problem. Navigation of the UAV was achieved by continuously sending local position messages to the autopilot via Mavros. The proposed system performed hovering above the target in three different stages: locate, descend, and hover. The system was tested in multiple trials, in simulations and outdoor tests, from heights of 10 m to 40 m. Results show that the system is highly reliable and robust to sensor errors, drift, and external disturbance.

## 1. Introduction

Development in Unmanned Aerial Vehicle (UAV) path planning [1,2,3], design [4,5,6], and remote sensing [7,8,9,10] has been an active field of research over the past 15 years. Several researchers have studied the possibility of using vision for navigation, target tracking, and landing site detection. A predictive navigation technique was developed for a wildlife detection scenario in [11]. In [12], a vision-based control system for a quadcopter for following a ground-moving target was demonstrated. Vision-based autonomous landing on a landing pad is presented in [13]. In most of the earlier work, the main focus has been on demonstrating the ability of the developed controllers and not on the reliability and robustness of the systems. Our motivation was to use a low-cost UAV system to remotely sense and find a target, and reliably descend and hover above the target with the purpose of closely inspecting it. The system can also be used for accurate and safe delivery of payloads or spot spraying applications in site-specific crop management.

**Figure 1:**
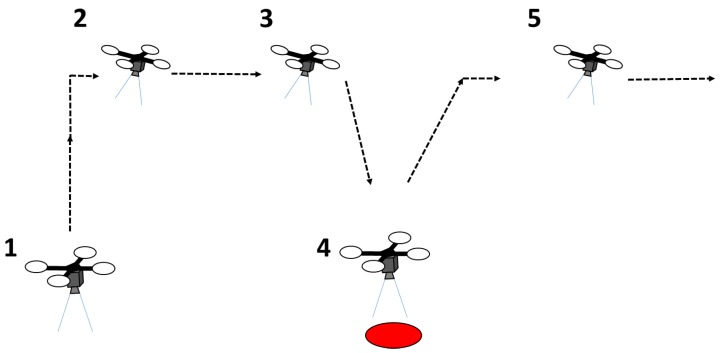
Typical mission performed by the multirotor UAV in a target finding and inspection mission.

Figure 1 illustrates the concept and a typical mission. Initially, the UAV has to take off (1). After takeoff, the UAV searches for a ground target (2). If a target is found, the UAV changes its original path (3) and hovers above the target for a few seconds for an action such as close inspection (4). After hovering, the UAV will fly to the next waypoint (5) and start to search for other targets. This paper focuses on target detection and navigation. A red-colored 0.2 m radius circular target was used in this study for illustrative purposes.

The system uses a modular system architecture and a vision-based algorithm with the observe, orient, decide, and act (OODA) decision loop [14] for searching and hovering above a target. The OODA loop is commonly used by military pilots during the decision making process. The OODA loop has also been used in other contexts such as modeling the development of intelligent agents [15,16]. Our results from multiple outdoor flight tests show that the proposed algorithm is reliable and robust regardless of sensor errors and external disturbances.

## 2. Related Work

Related work in the field of vision-based navigation includes work by Mathe and Busoniu [17] who reviewed vision and control for UAVs in the context of inexpensive platforms for infrastructure inspection. They defined UAVs under $1500, diameter under 1 m, and weight under 4 kg as inexpensive platforms. The authors grouped the existing work, including the use of more costly UAVs, into three groups: power line inspection, building monitoring, and railway inspection. A general technique used in this application is tracking lines in infrastructure. Araar and Aouf [18] demonstrated a power line inspection task using a quadrotor. The authors developed a classical image-based visual servoing controller and a partial pose-based visual servoing controller for this purpose.

Mathe et al. [19] presented the task of inspecting railway semaphores using a proportional controller based on the distance to the object and flying the UAV around a semaphore in a 4–5 m radius arc. However, the study did not consider the part of navigating the UAV from a high altitude to the railway semaphore.

In other related work, a robust marker tracking algorithm was presented for precise UAV vision-based landing in [20]. The authors employed a pinhole camera model to calculate the relative position of the UAV from the landing target. The study was limited due to simulation and estimation errors and disturbances such as wind that were not considered.

Vision-based landing on a moving vehicle was demonstrated in [21]. The authors used the relative distance between the UAV and the vehicle to generate velocity commands to the UAV. Therefore, extending this method to multiple target-finding scenarios is difficult. In [22], a target detection and tracking algorithm was discussed with the aim of landing the UAV on a target. The method proposed was effective only for detection and tracking with low disturbances. Furthermore, the method was only tested in simulation.

An open-source computer vision-based guidance system for landing and hovering the UAV on a target was proposed in [23]. The authors used simple geometry to estimate the target’s position and position control to guide the UAV towards the target. Their method was tested only with the UAV cruising a few meters (3–5 m) above the ground. Even though there is literature on the topic related to this work, this paper contributes to the literature by extending the technique to high altitude flights.

## 3. Hardware System

**Figure 2:**
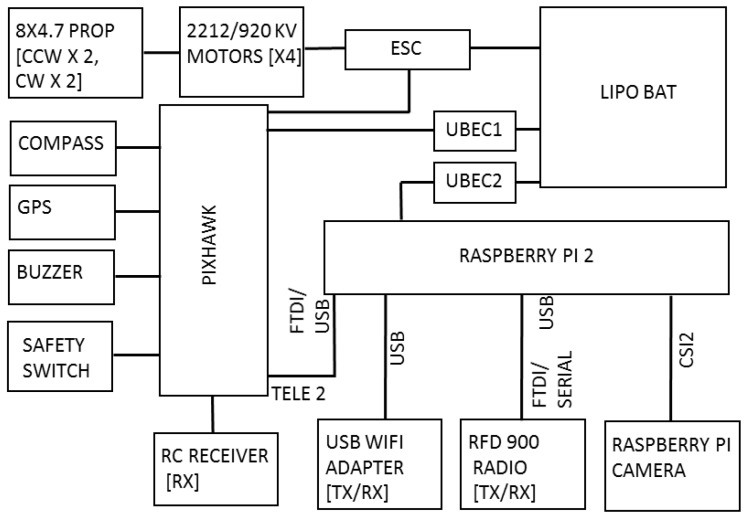
Hardware system architecture illustrating the hardware components and their interconnection.

**Figure 3:**
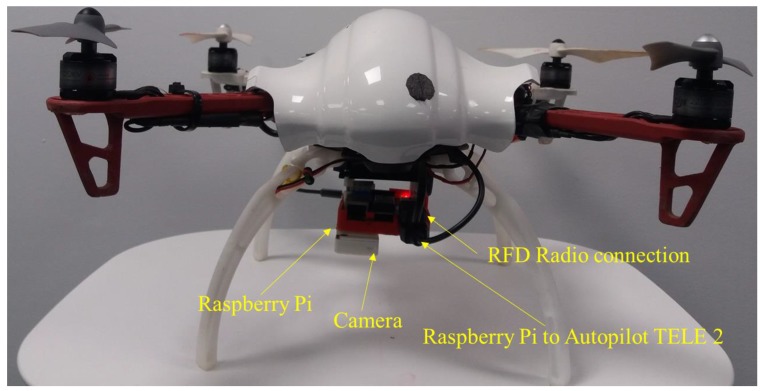
Hardware system.

The hardware system architecture is shown in Figure 2 and the complete hardware system is shown in Figure 3. A quadrotor UAV that uses the DJI 450 frame was developed for the purpose of this study. Four DJI 2212/920 KV motors and 8 **×** 4.7” propellers were used to produce thrust. The open source Pixhawk autopilot board was used as the main flight controller. The Pixhawk controller supports PX4 and APM firmware stacks. However, the PX4 firmware stack was used as it has more hardware support [24]. The Pixhawk autopilot board consist of gyroscopes, accelerometers, and a barometer as sensors. A ublox GPS + compass module was externally mounted for positioning. The GPS and compass modules were mounted externally and away from other electronics to reduce interference and increase accuracy. A Raspberry Pi 2 computer was attached to the UAV as an onboard computer to run the detection and navigation algorithm. Ubuntu 14.04 and ROS Indigo were installed on the onboard computer.

A downward-looking Raspberry Pi camera was attached to the UAV as an imaging sensor. This camera is capable of taking images with 640 × 480 resolution at 90 fps. A USB Wi-Fi adapter was connected to the Raspberry Pi computer for the purpose of debugging the code from the development PC. A terminal was configured for the onboard Ubuntu operating system to remotely access the UAV from the ground computer through a RFD 900 long-range radio. This terminal connection was used for monitoring flight log messages and initiating the autonomous flight. UBEC 1 and UBEC 2 were used to distribute the power to the Pixhawk and Raspberry Pi board, respectively.

## 4. Software System

**Figure 4:**
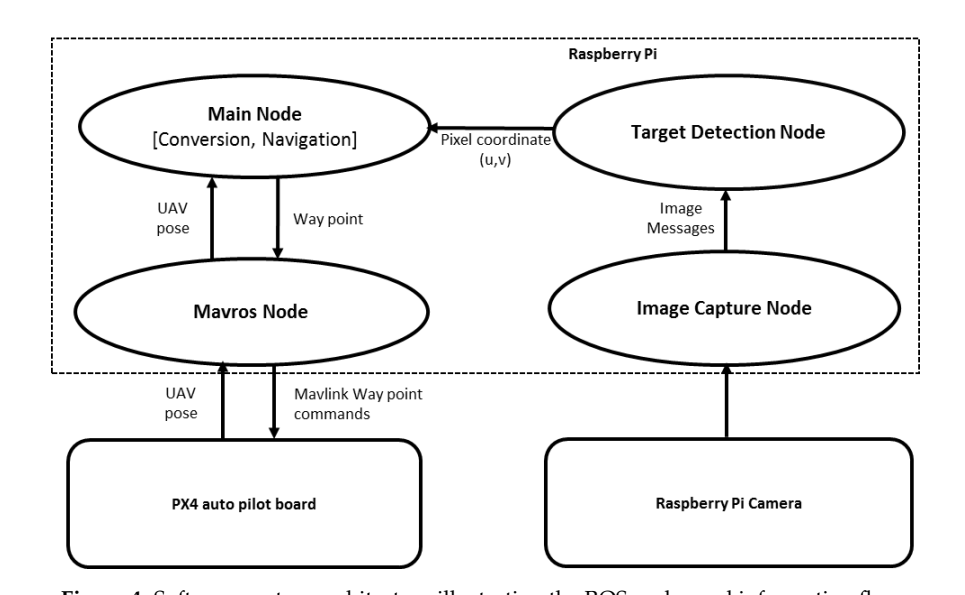
Software system architecture illustrating the ROS nodes and information flow.

The software system consists of four ROS [25] nodes (Figure 4): an image capture node that captures the images from the Raspberry Pi camera at a predefined frame rate; a target detection node that uses a modified version of the algorithm described in [26] for target detection; a main node that runs the main control loop of the system; and a Mavros node that provides communication between the ROS and PX4 autopilot.

The image capture node, target detection node, and the main node were developed in C++. Mavros is a prebuilt package available with ROS. OpenCV is used to capture images in the image capturing node. All the decision and guidance functions are performed by the main node. The following subsections describe in detail each function of the main node.

### 4.1. Conversion of 2D Image Coordinates to World Coordinates

**Figure 5:**
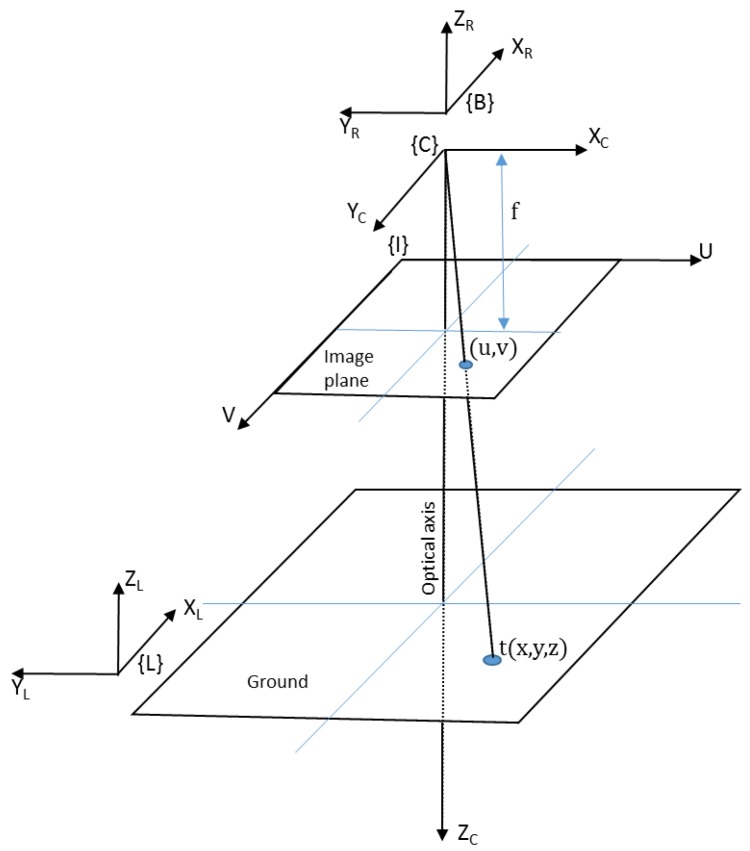
Pinhole camera model and coordinate frames used for converting the image target coordinates (u,v) into world coordinates (x, y, z).

The main node receives the center coordinates of the detected target from the target detection node as an input. This is then converted to the target location in the inertial frame by using the pinhole camera model (Figure 5). The coordinates of the target in world coordinates (inertial frame {L}) are given by Equation (1).

(1) t L=ξ LC . t C

The center of the target in the camera frame t C is given by Equation (2):

(2) t C=[xyz]=S R CK−1[uv1]

where *u* and *v* are pixel coordinates of the center of the target in image frame {I}. The projected pixel coordinates change with the rotation of the camera. Therefore, the rotation of the camera in its own frame R C is used for correcting the rotation. *K* is the camera matrix and is found by using the ROS camera calibration package as described in the tutorial [27]. *S* is an unknown constant. *S* can be solved by equating the last rows of the final matrix from the right side to the UAV’s flying height z.

The camera is directly attached to the UAV without any gimbal mechanism. Therefore, the camera rotation matrix R C is written as:

(3) R C=R(−θ)R(−ϕ)R(−ψ)

where ϕ, θ, and ψ are roll, pitch, and yaw angles of the UAV in the body frame {B}. The camera pose in inertial frame ξ LC is given by Equation (4):

(4) ξ LC =ξ  LB . ξ  BC

where ξ LB is the pose of the body frame {B} in inertial frame {L}. It is given by the position of the UAV. The camera pose in body frame ξ BC is dependent on the camera mounting arrangement. In our case, it can be written as a homogeneous matrix (Equation (5)):

(5) ξ BC=[0−100−100000−100001]

### 4.2. Navigation Algorithm

Navigation of the UAV is achieved by continuously sending the local position messages from the main node via the Mavros [28]. The proposed algorithm performs the task of hovering above the target for close inspection through three different stages: locate, descend, and hover.

The OODA loop makes different decisions based on this stage of the UAV. In the locate stage, the estimated target position *TP* (line 30) is used to move the UAV laterally to the target’s *x,y* position without descending (lines 32, 33 and 34). In the descend stage, the lateral deviation between the target’s position *TP* and the UAV’s position *CP* is calculated (line 37). If the deviation is within the tolerance *t*, the height of the multirotor is reduced by a predefined parameter *d_h_* (line 39). Otherwise, the locate stage will be repeated (lines 45 and 46). This process continues until the multirotor reaches the hovering height *h_h_* (line 38). This process happens in quick succession.

After reaching the hovering height, in the hover stage, if the distance between the center of the target and the center of the image in pixels is more than the tolerance value *g* (line 49), a proportional controller generates displacement vectors based on the pixel distance between the centers. These displacement vectors are used to adjust the position of the multirotor above the target (lines 50–54). In this stage, the camera field of view is very small. Therefore, if the target is out of the field of view, the last detected target pixel coordinate *CTL* is used for a timeout period *t_o_* to laterally move the UAV above the target (lines 61–66). If the UAV is exactly above the target, the position is maintained for *s* seconds (line 56). Finally, the UAV flies to the next waypoint (lines 71 and 72).

**Algorithm 1** Navigation Algorithm
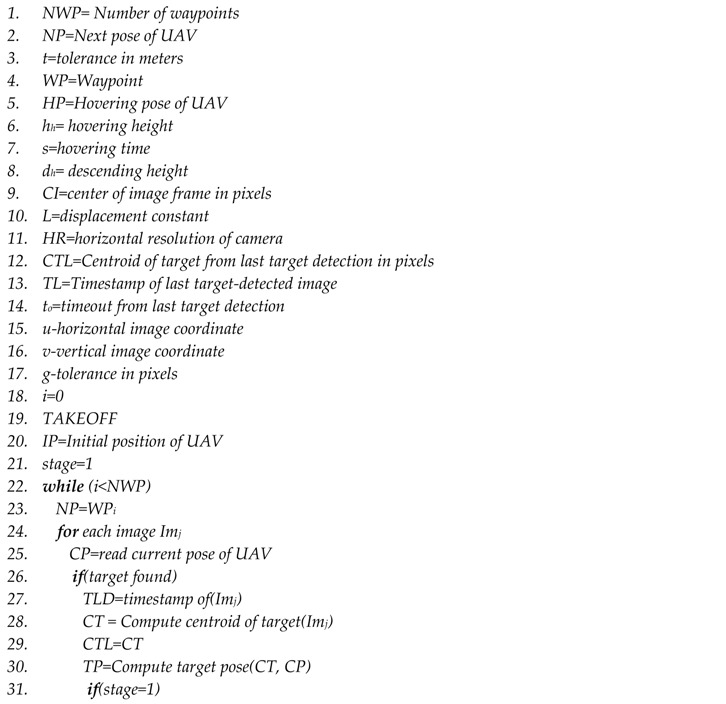

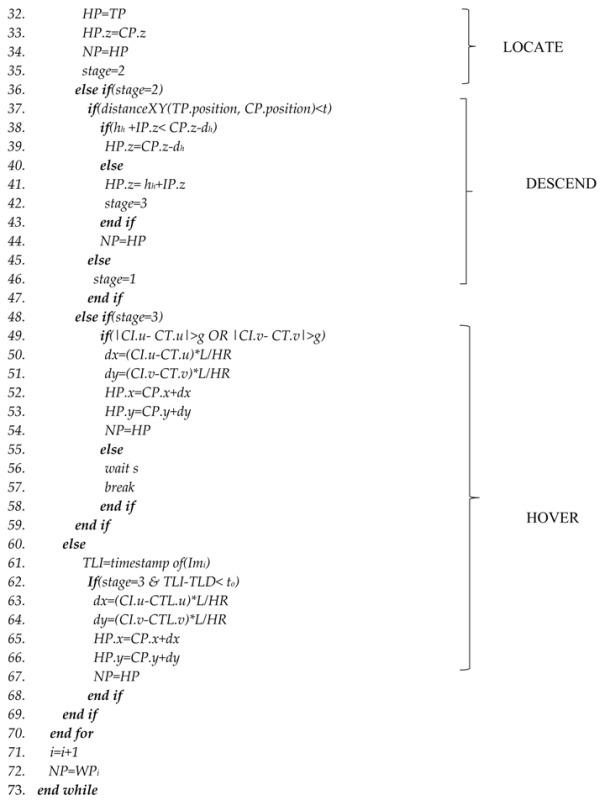


### 4.3. Target Detection Algorithm

A robust target detection is needed for the navigation algorithm to perform properly. Different target detection techniques were tested. The Hough circle method described in [26] was used to detect the target. However, when increasing the altitude, the detection became less reliable. The color detection method described in [29] *was* also used and gives reliable detection at high altitudes; however, false positives were observed at low heights. A detection algorithm (Algorithm 2) was developed by combining both techniques.

**Algorithm 2** Target Detection Algorithm*1.****for** each image Im_i_**2.* *u=v=−1**3.* *Im_i_=preprocess(Im_i_)**4.* *binaryImg= threshold (Im_i_, colorRange)**5.* *binaryImg=postProcessing(binaryImg)**6.* *[u,v]=findHoughCircleCenter(binaryImg)**7.* ***if**(u<1 OR v<1)**8.*  ***if**(moment00(binaryImg)>0)**9.*   *[u,v]=findCentroidOfBlob(binaryImg)**10.*  ***end if****11.* ***end if****12.****end for***

In Algorithm 2, a binary image is created for red-colored blobs using a threshold operation (line 4). The center of the target is found using the OpenCV HoughCircle function (line 6). If no Hough circle is found, but blobs exist (lines 7 and 8), then the image moments are used to find the centroids of the blob (u,v) in pixels (line 9).

(6) $$u=\frac{m_{10}}{m_{00}}$$

(7) $$v=\frac{m_{01}}{m_{00}}$$

where $m_{00}$ is 0th moment and $m_{10}$ and $m_{01}$ are the first-order moments.

A ground-based test was conducted where the target was moved by walking slowly away from the camera and the detection of the new algorithm was observed. The distance between the camera and the target was measured. From this test, the range of the target detection algorithm was determined as 88 m under bright sunlight.

## 5. Experiments

Simulated and real flight experiments were conducted to validate the developed system. There were two goals for the experiments. The first goal was to descend and hover the UAV reasonably close to the target with the purpose of inspecting the target. To achieve this goal, a criterion was defined such that if the UAV could hover above the target within an area of 1 m from the center of the target in the inertial *xy* plane and within 2.5 m above the ground, then the mission would be considered as successful. The second goal was to test the reliability of the system. To achieve this goal, tests were repeated multiple times with different parameters. The Experimental Scenario subsection describes the details.

### 5.1. Experimental Scenario

**Figure 6:**
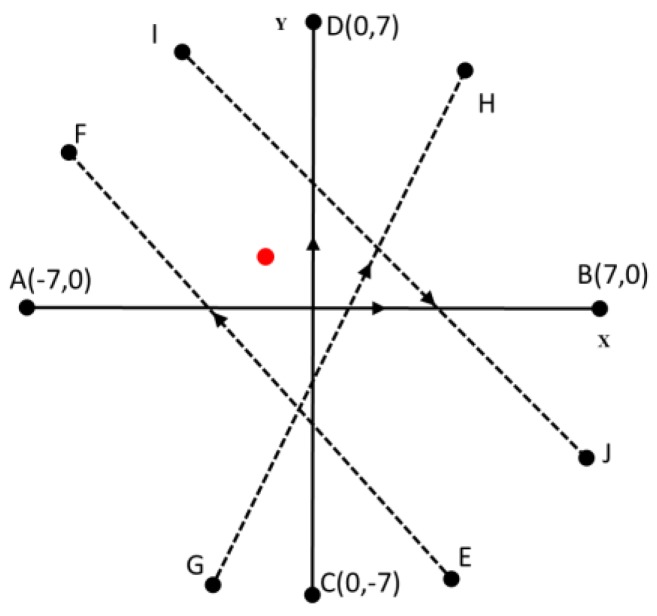
Typical flight paths used in each height of the experiment and an example target location (red circle). Waypoints are indicated by (•).

Figure 6 shows the experimental scenario used for both simulation and field tests. The UAV had to take off and move from one waypoint to another waypoint while scanning for a red circle target on the ground. A 0.2 m radius red-colored circular target was placed on the ground between the waypoints.

The UAV was flown at different heights ranging from 10 to 40 m. Each height was repeated five times. When repeating the heights, the coordinates of the waypoints were changed to ensure the ability of the system with different flight scenarios (Figure 6). The target was also placed randomly along the path, to the right or left sides of the flight path, but so the target was visible to the UAV when the UAV flew along the flight path. In Figure 6, letters indicate the waypoints and arrows indicate the flight paths used. Paths AB and CD were flown according to the coordinates shown in the diagram. Paths similar to EF, GH, and IJ were chosen randomly.

### 5.2. Simulation Experiments

Refining and testing the system is mandatory before doing any field flight test. Therefore, a Software in the loop (SITL) simulation environment was set up using the Gazebo robotics simulator [30] and the PX4 firmware for the Pixhawk autopilot hardware. A quadrotor model and simulated world were created to closely resemble the real experimental environment. Real images captured from the field test were also used in modeling the simulation environment. A downward-looking simulated camera model was attached to the simulated quadrotor. Simulated camera parameters were set to the real Raspberry Pi camera’s parameters used in the real test. A very high frame rate increases the image processing load in the Raspberry Pi computer and may introduce additional delays. Therefore, the camera frame rate was set to 10 Hz in the real test as well as in the simulation. Horizontal flying velocities of the multirotor were kept similar to the field tests by setting the PX4 parameters.

**Figure 7:**
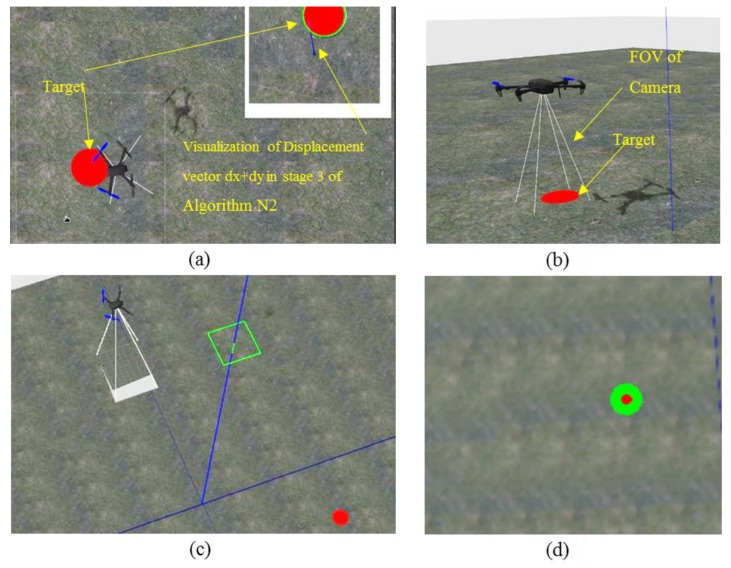
Simulation environment, (top) Unmanned Aerial Vehicle (UAV) is approaching the target. (**a**) Plane view; (**b**) 3D view; (**c**) UAV is flying at 20 m height; (**d**) target detection from 20 m height.

Figure 7a shows the UAV approaching the target in a plane view and Figure 7b shows the UAV above the target at hovering height. Figure 7c shows the UAV flying at a height of 20 m and Figure 7d shows target detection using the simulated camera where the UAV is at a height of 20 m.

### 5.3. Flight Test Experiments

**Figure 8:**
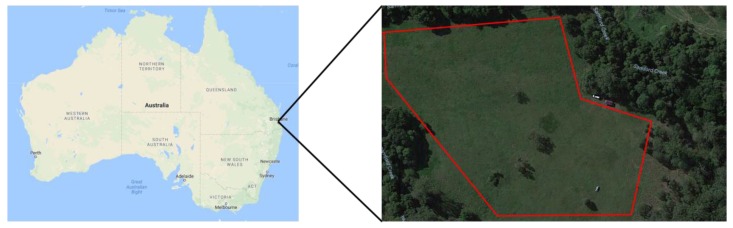
The marked area shows the test site (Samford Ecological Research Facility, Queensland University of Technology (QUT), Queensland, Australia).

**Figure 9:**
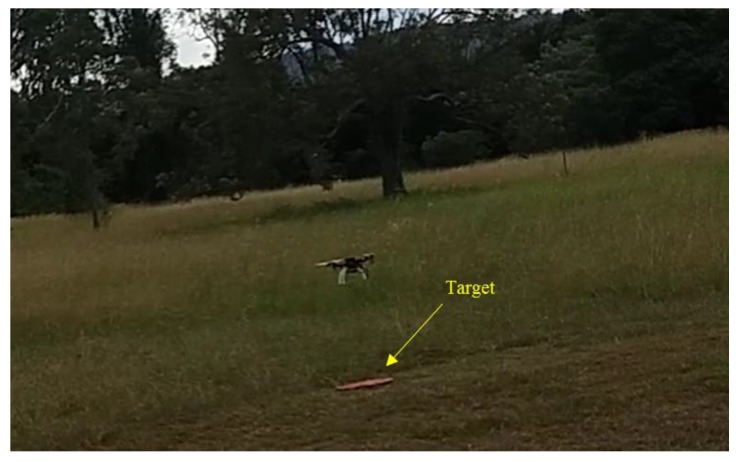
UAV is hovering above the target at hovering height (simulation of close inspection).

Flight tests were conducted at Queensland University of Technology’s (QUT’s) Samford Ecological Research Facility (SERF), Queensland, Australia on different days, including cloudy and overcast days, over a four-month period. The same experimental scenario described in Figure 6 was followed. Figure 8 shows the test site and the mission boundaries. Figure 9 shows the UAV hovering above the target during a field test.

## 6. Results and Discussion

All of the simulated flight experiments were successful. In the real flight test, the overall success rate was 92%. Two flight tests failed: one at 15 m and one at 30 m altitude. Our analysis of the failed tests showed that the timeout period of 1 s had expired in the hover stage of the navigation algorithm. Therefore, the UAV had lost track of the target. However, we believe that experimentally adjusting the timeout period to a more suitable value can reduce the problem. Table 1 summarizes the results of the simulation and field tests.

**Figure 10:**
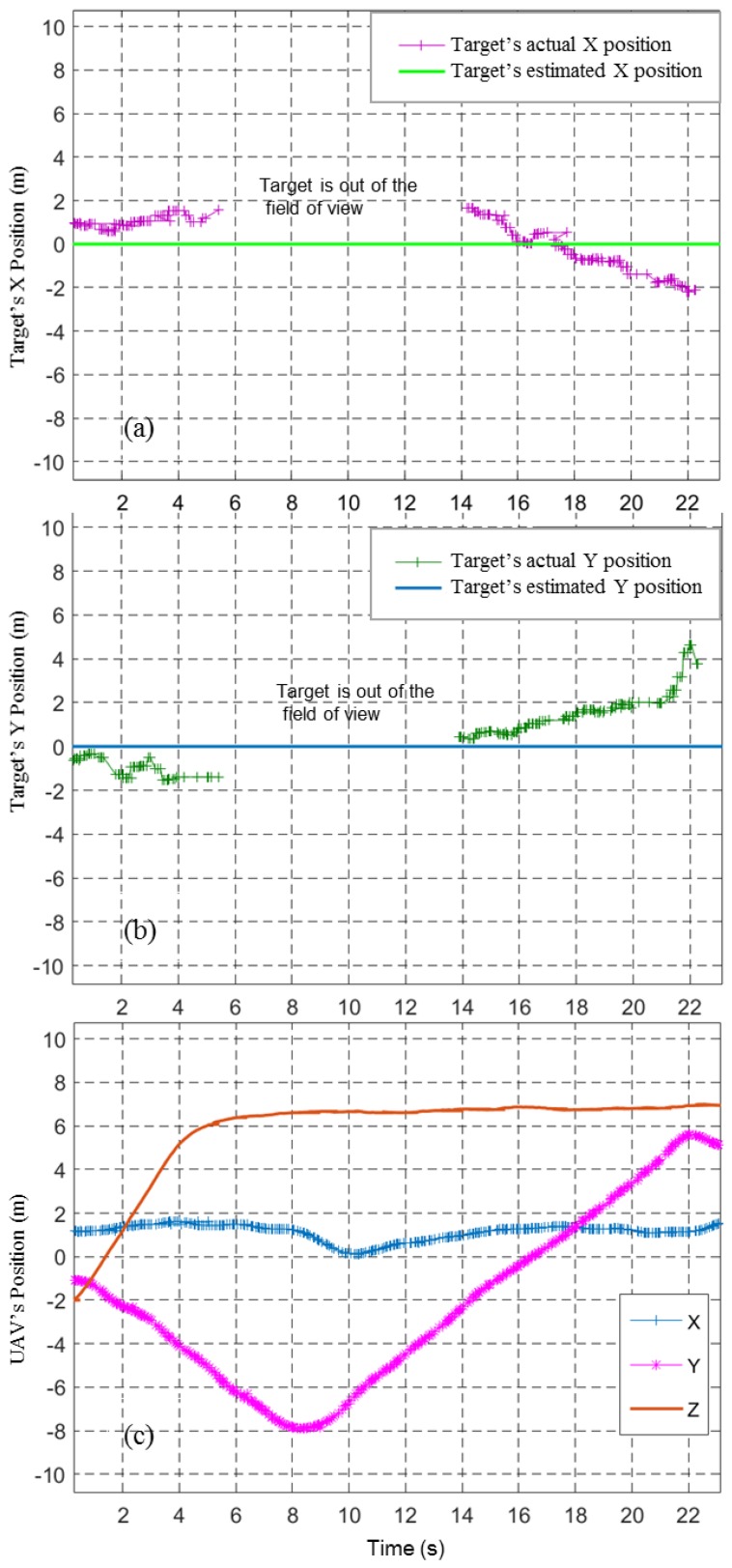
Image-based target position estimation and the actual position of the target from a 10 m height flying mission. (**a**) Target’s actual and estimated X positions; (**b**) Target’s actual and estimated Y positions; (**c**) UAV positions.

The task performed by the system is simple if all the sensor measurements are ideal and there are no external disturbances. However, target position estimations in the real world have considerable errors and uncertainty. Figure 10 shows these errors clearly. It shows the recorded target’s X and Y position estimates and the UAV position when the UAV took off and flew at a height of 10 m from waypoint A to waypoint B without descending to the target.

**Figure 11:**
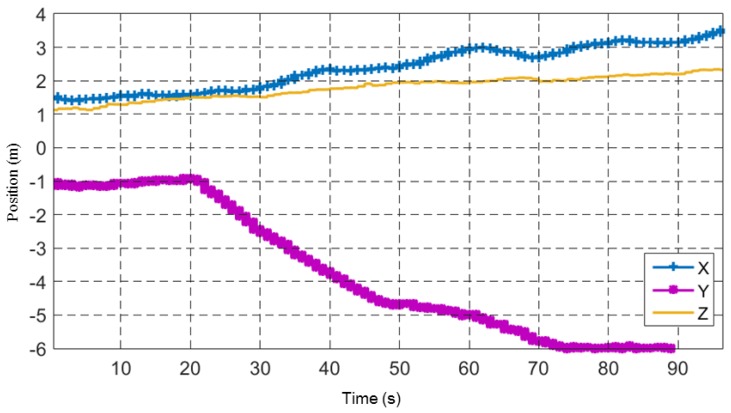
A plot of the autopilot’s position estimation drift when the UAV is stationary on the ground.

Nisi and Menichetti [31] listed and gave a good discussion of the error sources in their task of geo-referencing photovoltaic arrays using UAVs. Among the error sources discussed by Nisi and Menichetti are barometric altimeter resolution, UAV velocity, target height, lens distortion, GNSS accuracy, and imaging sensor resolution, which are also all applicable to our task. Moreover, experimental data collected showed considerable drift in the autopilot’s inertial frame {L} (Figure 11). The UAV’s position data shown in Figure 11 was recorded while the UAV was resting in a static position on the ground. The plot shows significant drift in the inertial frame of the UAV.

Further to the error sources discussed in [31], the target position estimation method assumes that the camera is in perfect alignment with the UAV’s body frame and that both the camera frame and the body frame have the same origin. There are practical limitations in aligning the camera and the Inertial Measurement Unit (IMU) of the UAV. Other sources such as errors in synchronization of data from different sensors (camera, IMU) and camera vibration also contribute to the overall error in target position estimation.

**Figure 12:**
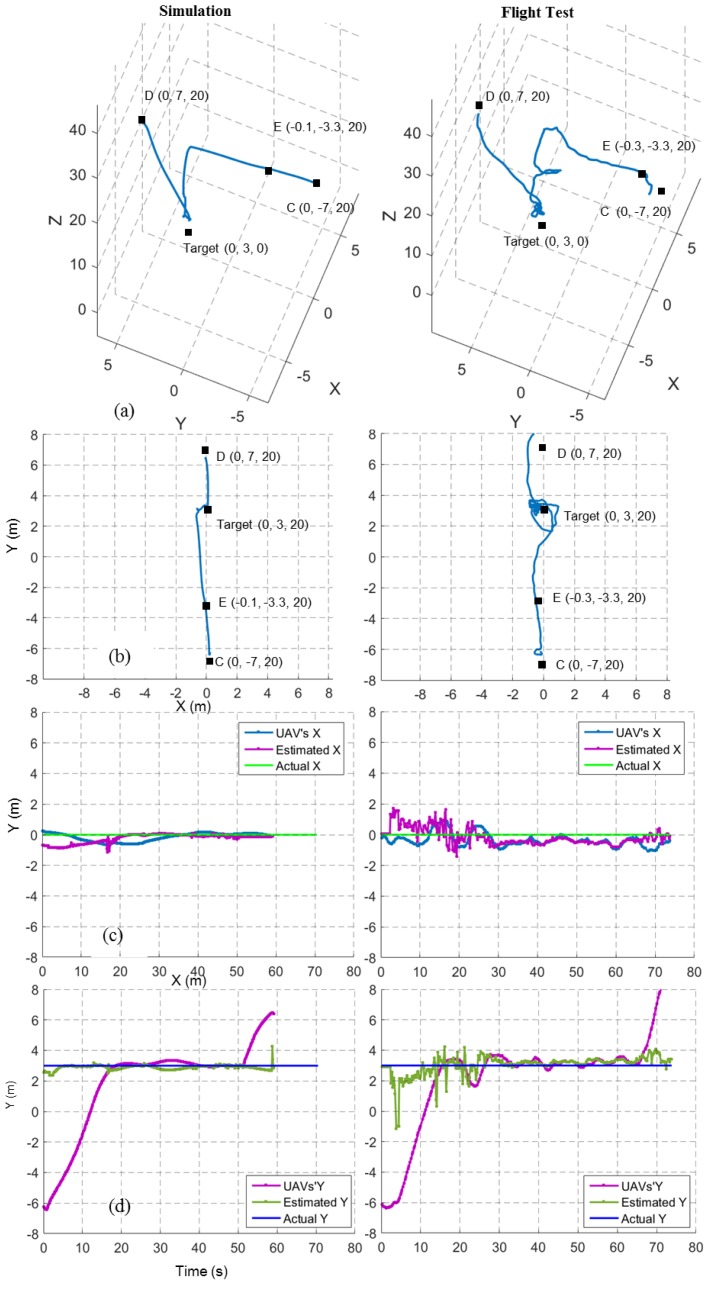
Flight trajectories from a 20 m simulated and a real target finding and hovering missions. Left column shows simulated results and the right column shows field test results. (**a**) 3D View; (**b**) top view; (**c**) X coordinates of UAV position, estimated target position and real target position; and (**d**) Y coordinates of UAV position, estimated target position and real target position.

Figure 12a,b shows the 3D view and top view of the flight trajectories for a target finding and hovering mission at a 20 m height for both simulation and field tests. Here, the UAV was expected to move from waypoint C to waypoint D. The point E indicates the decision point where the system decided to move towards the target in the *x,y* before descending. The path in the field test, compared to the simulation, took a small spiral shape. This was mainly because of the image-based target position estimation errors and external disturbances such as wind. The top view of the field test shows a slight difference between the target’s position and the hovering position. The drift in the inertial frame {L} was the cause of this effect. Figure 12c,d show X and Y coordinates of the UAV, estimated target position and real target position for the same 20 m target finding and hovering mission.

**Figure 13:**
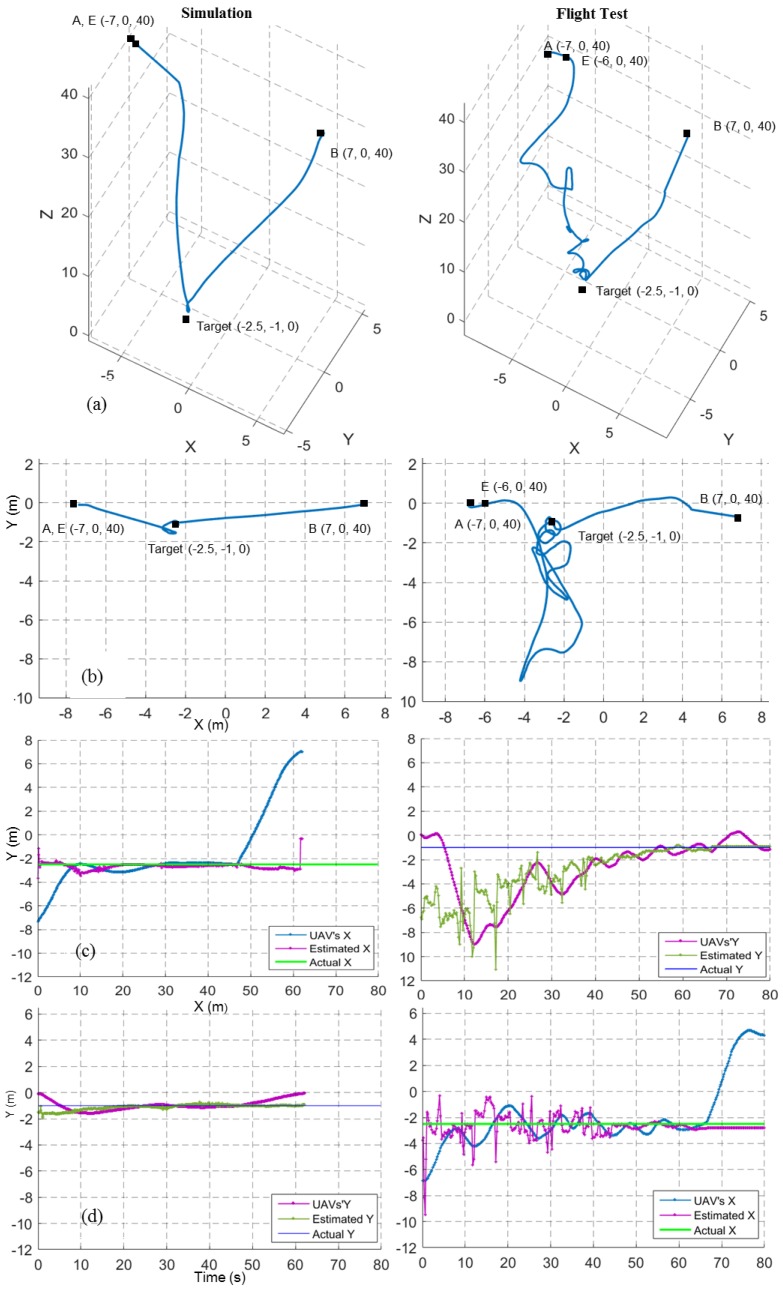
Flight trajectories from a 40 m simulated and a real target finding and hovering missions. Left column shows simulated results and the right column shows field test results. (**a**) 3D View; (**b**) Top View; (**c**) X coordinates of UAV position, estimated target position and real target position; (**d**) Y coordinates of UAV position, estimated target position and real target position.

Figure 13 shows the results for a target finding and hovering mission at a 40 m height. Here, the UAV was expected to move from waypoint A to waypoint B. The point E indicates the decision point where the system decided to move towards the target. Compared to the 20 m flight test (Figure 12b, right), the 40 m flight test (Figure 13b, right) shows an increase of a spiral-like path when the UAV descended. An increase in the image-based target position error (Figure 13c,d, right) was the main reason for this.

In contrast, the 40 m simulation results show less error, similar to the 20 m simulation results. One possible reason might be that all the sensors in the simulation were ideal, except the IMU. Though the position estimation error increased with height in the flight test, the UAV could still compensate for the error and reach the target.

Infrastructure inspection with inexpensive UAVs is a growing field. Researchers have explored manually positioning the UAVs at a workable distance from the target. However, a fully autonomous system with the capability of finding and approaching the target is desirable. For instance, in railway infrastructure inspection tasks, the line tracking techniques discussed in earlier papers can be used to fly the UAV along the railway track at a 50–100 m altitude and the technique presented here can be used to approach an identified target and do a detailed inspection.

Moreover, purely image-based techniques without position estimation such as image-based visual servoing are useful in vision-based landing tasks. The technique may have limitations when multiple targets have similar features or in detecting distinguishable features when the targets are very far from the UAV. Generally, researchers have used relatively large targets with uniquely distinguishable features for vision-based landing tasks, where the challenges due to sensor errors and disturbances are less prevalent.

The navigation algorithm presented here tested with a reliable detection (>90%). However, target detection algorithms may be less reliable in real applications. Performance of the navigation algorithm might be affected when the target detection is unreliable. Further research is needed to make the algorithm robust for such cases.

## 7. Conclusions

In this paper, a system architecture and a reliable and robust algorithm for inspection of the ground target using an inexpensive multirotor UAV system is presented. Image-based target position estimation was used to guide the UAV towards the target. In an ideal sense, the task was very simple. However, uncertainties and errors introduced by the low-cost onboard sensors and disturbances in the outdoor environment present significant challenges to reliably perform the task. The authors applied a high-level decision making approach with the observe, orient, decide, and act (OODA) loop to address the problem. The results from multiple field tests show that the proposed algorithm is 100% reliable in simulation and 92% in real experiments and robust to sensor errors, drift, and external disturbances.

Future work will focus on extending the system for multiple targets. Multiple target detection and inspection needs real-time detection and tracking of each target. This is a more challenging task owing to the limited onboard computation power that is available with the inexpensive UAV system. Moreover, path planning and decision making are also important to optimally visit the targets with the limited battery capacity.

A video of the flight test can be found at the link https://youtu.be/6w_OFScBWIg.

## Figures and Tables

**Table 1:** Results of simulations and flight tests.

Height (m)	Success Rate (Simulation)	Success Rate (Flight Test)
10	100% (5/5)	100% (5/5)
15	100% (5/5)	80% (4/5)
20	100% (5/5)	100% (5/5)
30	100% (5/5)	80% (4/5)
40	100% (5/5)	100% (5/5)
